# Impact of the socioeconomic status on the severity and outcome of community-acquired pneumonia among Egyptian children: a cohort study

**DOI:** 10.1186/2049-9957-3-14

**Published:** 2014-04-24

**Authors:** Seham Fathy Abdel Hameed Azab, Laila M Sherief, Safaa H Saleh, Wafaa F Elsaeed, Mona A Elshafie, Sanaa M Abdelsalam

**Affiliations:** 1 Faculty of Medicine, Zagazig University, Egypt, 18 Omar Bin Elkhattab St, Al Qawmia, Zagazig City, Al Sharqia Governorate, Egypt

**Keywords:** Socioeconomic, Community acquired pneumonia, Children

## Abstract

**Background:**

Community-acquired pneumonia (CAP) is one of the five leading causes of death among children in developing countries, accounting for approximately three million deaths per year. Identification of the modifiable risk factors of CAP may help to reduce the burden of this disease. In this study, the impact of the socioeconomic status (SES) on the severity and outcome of CAP among Egyptian children was studied.

**Methods:**

This was a prospective longitudinal cohort study which included 1,470 children diagnosed with CAP, aged two to 15 years (median age 5.4 years). The diagnosis of CAP was based on clinical and radiological findings. A structured questionnaire and the patients’ medical records were used for the data collection. The subjects were divided into two groups: mild and severe CAP. Social and demographic variables were compared, and a multivariate logistic regression analysis was performed.

**Results:**

The multivariate analysis showed that a low maternal education level (OR: 3.8; 95% CI: 2.12 –6.70; *P* = .0001), unavailability of adequate medical care (OR: 3.1; 95% CI: 1.99 –4.88; *P* = .0001), a low family income (OR: 2.2; 95% CI: 0.99 –4.78; *P* = .047), and parents’ smoking habits (OR: 2.0; 95% CI: 1.15 –3.55; *P* = .014) were significant independent predictive risk factors for severe CAP among Egyptian children.

**Conclusion:**

Public health measures against these socio-demographic risk factors should be identified as priorities in order to help reduce the disease burden of deaths from severe CAP among Egyptian children.

## Multilingual abstracts

Please see Additional file 1 for translations of the abstract into the six official working languages of the United Nations.

## Background

Community-acquired pneumonia (CAP) is the most severe form of an acute respiratory infection, accounting for 80% of all deaths from such an infection [1].

There are approximately 150 million cases of childhood CAP reported each year [2]. Although death from CAP is rare in industrialized countries, lower respiratory tract infection is one of the leading causes of childhood mortality in developing countries [3].

Despite progress in life-support measures and antimicrobial therapy, the mortality of severe CAP has not varied since the mid-1990s, suggesting that other factors are of crucial importance in the evolution of this infection [4]. The socioeconomic status (SES) of the patient and his/her family is one of the most robust social factors associated with health, but the dynamics of how the SES affects children’s health over time remains unclear [5]. Factors associated with CAP-related complications including the age of the child, maternal age, the level of maternal education, acute malnutrition, and lack of breastfeeding have been studied [6].

Over the past two decades, there have been several attempts to investigate the relationship between socio demographic risk factors and severe pneumonia in young children, but few reports have proven whether this relationship actually exists [6,7]. The lack of epidemiological studies from developing countries makes it difficult to develop effective intervention strategies that may help to reduce the overall burden of this disease. The aim of our study is to detect the impact of the SES on the severity and outcome of community CAP among Egyptian children.

## Methods

This was a prospective longitudinal cohort study performed at the Zagazig University Children’s Hospital and the Outpatient Clinics at the same hospital from August 2009 to June 2013. In the four 4-year period, 1,470 children diagnosed with CAP (891 males and 579 females) were enrolled in the study. The age of the patients ranged from two year to 15 years (median age 5.4 years). The diagnosis of pneumonia was based on clinical findings (fever, cough, chest pain, and difficulty breathing); physical examination findings (tachypnea, chest retraction, and decreased breath sounds or rales); and radiological findings [8]. Chest radiographs were evaluated by a radiologist trained in reading and interpreting radiographs according to the World Health Organization’s (WHO) guidelines [9]. Pneumonia was considered as community-acquired if the patient had no history of hospitalization during the two weeks prior to admission [10].

The exclusion criteria were:

1. Neonate (<four weeks of age) and children less than two years of age;

2. Clinical diagnosis of bronchiolitis;

3. Children with a pre-existing lung disease, particularly asthma;

4. Children with a congenital heart disease, or a chronic liver or kidney disease;

5. Postoperative children;

6. Patients hospitalized in the past 30 days; 7. Patients with immunodeficiency; 8. Patients receiving treatment with corticosteroids equivalent to 20 mg/d of prednisolone for more than 14 days; and 9. Patients who have undertaken chemotherapy or immunosuppressive drugs in the past 60 days.

All patients were subjected to the followings:

1) An adequate history/background check, placing special focus on the child’s birth weight, immunization, history of respiratory infection two weeks prior to admission to hospital, and feeding practice (this included either never being breastfed or being breastfed) and symptoms such as fever, cough, difficulty breathing, chest pain, wheezing, or cyanosis.

2) A thorough clinical examination with particular attention paid to anthropometric measurements, and the presence and location of findings from the physical examination, such as retractions, grunting, focal decreased breath sounds, rales, and wheezing.

3) Laboratory investigations, which included:

3.i. A complete blood count (CBC) including blood indices, serum ESR 1st hour, C-reactive protein (CRP), blood PH, serum Na level, and liver function and kidney function tests.

3.ii *Sputum culture:* All children were categorized as having either positive or negative sputum culture for bacteria.

3.iii *Blood culture:* All children categorized either positive or negative blood culture for bacteria.

4) Radiological studies, which included: A chest X-ray was conducted to determine the type of pneumonia the child had, which was either classified as lobar, interstitial, or bronchopneumonia.

5) Structured questionnaires and the patients’ medical records were also used for the data collection. The questionnaire was filled out by parents or caregivers and included information regarding the socio-demographic profile of the enrolled subjects. The main variables were the level of the parents’ education, their occupation, the family income, the family domain, the family possession domain, availability of adequate medical care, and housing conditions (residence, number of children in the household, parents’ smoking habits, main source of drinking water, i.e., protected or unprotected). The socioeconomic status (SES) of enrolled CAP cases was classified into two groups, as according to the classification categorized by El-Gilany et al. [11]: **Group 1** (n = 1125): CAP cases with a low SES status. **Group 2** (n = 345): CAP cases with a high SES status.

Patients were further subdivided according to the severity criteria sourced from the management guidelines of the British Thoracic Society [10]. Any of the following led to a classification of ‘severe’: tachypnea (RR >50 for children > one year old), dyspnea, oxygen saturation <93%, oxygen given, nasogastric feeds, intravenous fluid infusion, septicemia, empyema, high dependency, or intensive care. A ‘mild’ classification was given to those who were immediately discharged or stayed in hospital for <3 days, and were not given any oxygen, or any intravenous or nasogastric feeds.

Patients were subjected to clinical, laboratory, and radiological follow-ups to detect any improvements. Follow-ups were terminated when children no longer exhibited any symptoms or signs, and were discharged from hospital. The period of admission was categorized to < one week or ≥ one week.

### Ethics

Informed parental consent was obtained for all those deemed to be eligible to participate in the study. The study was conducted according to the rules of the Local Ethics Committee of the Faculty of Medicine, Zagazig University, Egypt.

### Statistical methods

The Statistical Package for Social Sciences (SPSS) version 19 was used for the data analysis. All values are expressed as mean ± SD. Chi-Square and Fisher’s exact tests were used to compare the proportions. A binomial logistic regression analysis was also performed, in which severe CAP was considered as the dependent variable and the socioeconomic variables including the level of parents’ education, their occupation, the family income, availability of medical care, and housing conditions were the independent variables. *P* < 0.05 was considered significant.

## Results

There were non-significant differences between the study groups in categories such as age and gender (*P > 0.05*), however, urban residency was significant for group 2 (*P < 0.01*) (see Table 1). More than half (642 [57%]) of group 1 cases had a good immunization record and 765 (68%) of these had an average birth weight. In addition, 609 (54%) of the cases in the same group were, or still are, being breastfed, meanwhile 516 (46%) cases were never breastfed. As expected, 304 (27%) of group 1 cases were malnourished compared to 17 (5%) of group 2 subjects. Poor immunization, a low birth weight, an artificial feeding practice, and malnutrition were more common among group 1 cases with a statistically significant difference between the studied groups (all *P < 0.01*; see Table 1).

**Table 1:** Demographic data of studied subjects

		**Group 1 (n = 1125)**	**Group 2 (n = 345)**	***P***
*Age, median* (Range), years		5.4 (2–15)	5.6 (2–14.5)	> 0.05^a^
*Male/female*		675/450	216/129	> 0.05
*Residence*, *N* (%)	Urban	417(37)	249(72)	*<* 0.01
	Rural	708(63)	96(28)	
*Immunization*, *N* (%)	Good	642(57)	336 (98)	*<* 0.01
	Poor	483(43)	9(2)	
*Birth weight*, *N* (%)	Average	765(68)	300(87)	*<* 0.01
	Low	360(32)	45(13)	
*Nutrition status, N* (%)	Average	821(73)	328(87)	*<* 0.01
	Malnourished	304(27)	17(5)	
*Feeding practice, N* (%)	Was or still breast feeder	609(54)	261(76)	*<* 0.01
	Never breast feeder	516(46)	84(24)	

*Chi-square test* was used for comparisons. *P* value < 0.05 indicates a significant difference. ^a^*Mann–Whitney U test*.

Of note, except for septicemia which was not observed in group 2 cases, both findings from the history/background check (cough, difficulty breathing, chest pain) and findings from the physical examination (tachypnea, focal rales, focal wheezing, triage temperature, and oxygen saturation%) did not show significantly different presentation between the studied groups (all *P > 0.05*); see Table 2).

**Table 2:** Clinical data of studied subjects

		**Group1 n (%)**	**Group2 n (%)**	***P***
*Cough*		1047(93)^a^	312(90)	> 0.05
*Difficulty breathing*		978(87)	288(83)	> 0.05
*Chest pain*		528(47)	52(45)	> 0.05
*Age specific tachypnea*		405(36)	132(38)	> 0.05
*Chest auscultation*	Focal rales	483(43)	135(39)	> 0.05
	Focal wheezing	327(29)	102(30)	
*Fever*	Temperature >38.5°C	819(73)	237(69)	> 0.05
	Temperature <38.5°C	306(27)	108(31)	
*Oxygen saturation%, N (%)*	93–100	933(83)	295(85)	>0.05
	<92	192(17)	50(15)	
septicemia *#*		39(3.4)	0(0)	*<* 0.01

*Chi-square test* was used for comparisons. *P* value < 0.05 indicates a significant difference.

*#Fisher’s exact test*.

^a^Values in parentheses are percentages.

Our data showed that severe CAP was more common among group1 cases (439 [39%]) compared to group 2 cases (77 [22%]) (*P < 0.01*).

Table 3 shows the characteristics of the two subgroups with mild (n = 954) and severe (n = 516) CAP, as well as the socioeconomic risk factors potentially causing severe CAP. A young maternal age, a low maternal education level, parents’ smoking habits, rural residency, a low family income, and unavailability of adequate medical care were significantly associated with the risk of severe CAP (all *P < 0.01*), but parents’ occupation and housing conditions were not (*P > 0.05*; see Table 3).

**Table 3:** Socio-demographic variables and severity of CAP among studied subjects

		**Mild CAP cases**	**Severe CAP cases**	***P***
		**(n = 954)**	**(n = 516)**	
*Maternal age*	≤ 15 years	258(27)^a^	318(62)	*<* 0.01
	> 15 years	696(73)	198(38)	
*Maternal education level*	No or 1ry education	201(21)	345(67)	*<* 0.01
	2ry education or beyond	753(79)	171(33)	
*Maternal work*	House wife	496(52)	252(49)	*>* 0.05
	Worker	458(48)	264(51)	
*Father work*	Non worker	57(6)	36(7)	*>* 0.05
	Private worker	498(52)	248(48)	
	Gov. worker	399(42)	232(45)	
*Family income (Egyptian pounds/month)*	<1000 p/m	96(10)	369(71)	*<* 0.01
	1000-3000 p/m	144(15)	117(23)	
	>3000 p/m	714(75)	30(6)	
*Residence*	Urban	639(67)	138(27)	*<* 0.01
	Rural	315(33)	378(73)	
*Water supply*	Protected	534(56)	279(54)	*>* 0.05
	Unprotected	420(44)	237(46)	
*No. of children in the house*	One child	180(19)	87(17)	*>* 0.05
	> one child	774(81)	429(83)	
*Sharing bedroom*	One person	155(16)	67(13)	*>* 0.05
	> one person	799(84)	449(87)	
*Smoking habit of any parents*	No	696(73)	78(15)	*<* 0.01
	Yes	258(27)	438(85)	
*Medical care*	Poor	123(13)	393(76)	*<* 0.01
	Good	831(87)	123(24)	

*Chi-square test* was used for comparisons. *P* value < 0.05 indicates a significant difference.

^a^Values in parentheses are percentages.

All variables with *P*-values less than 0.25 were included in the multivariate analysis.

The final multiple logistic regression model indicates that a low maternal education level (OR: 3.8; 95% CI: 2.12 –6.70; *P* = .0001), unavailability of adequate medical care (OR: 3.1; 95% CI: 1.99 –4.88; *P* = .0001), a low family income (OR: 2.2; 95% CI: 0.99 –4.78; *P* = .047), and parent’s smoking habits (OR: 2.0; 95% CI: 1.15 –3.55; *P* = .014), were significant independent predictive risk factors for severe CAP among the studied children in decreasing order of odds ratio (all *P < 0.05*; see Table 4).

**Table 4:** Multiple logistic regression analysis of socio-demographic risk factors for severe CAP among studied subjects

**Risk factor**	***Coefficient***	***P value***	***Odds ratio***	***95% CI***^***1***^
*Maternal age*	.031	.174	1.031	.987-1.078
*Maternal education level*	-1.327	.0001	3.8	2.12 –6.70
*Smoking habit of any parents*	.701	.014	2.0	1.15 –3.55
*Residence*	.082	.907	.921	.230-3.685
*Family income*	-0.778	.047	2.2	0.99 –4.78
*Medical care*	-1.1372	.0001	3.1	1.99 –4.88

^1^CI indicates confidence interval.

**Figure 1:**
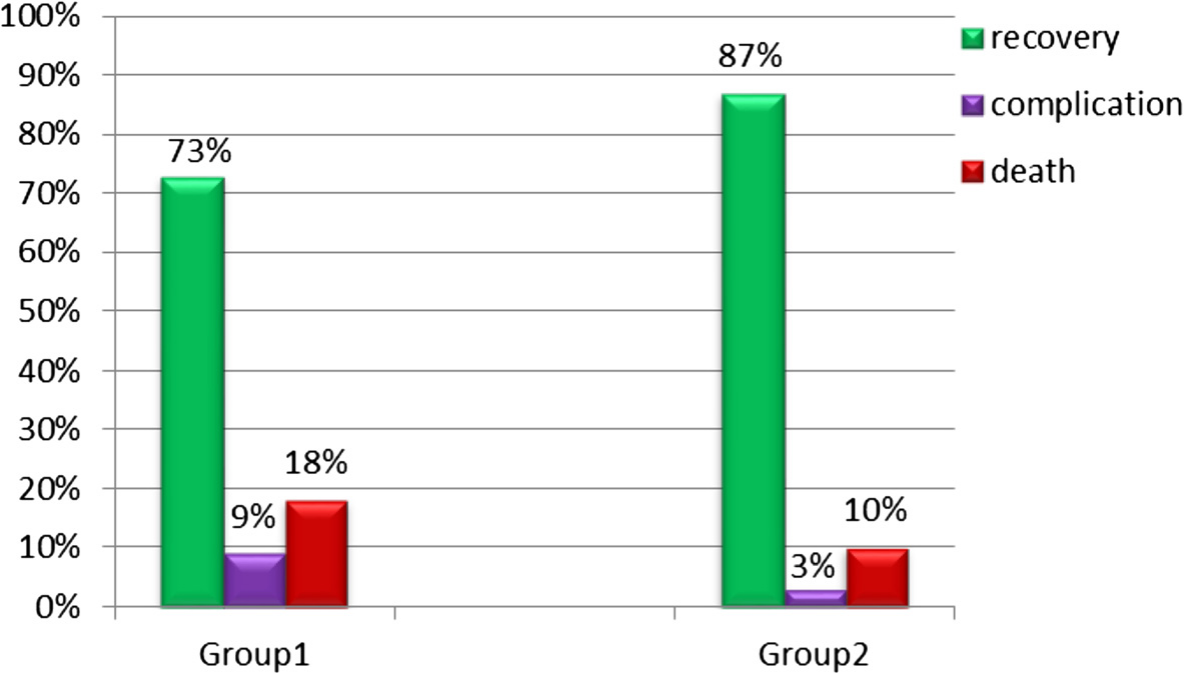
Outcome of CAP among the studied subjects (P < 0.05).

Our data showed that 822 (73%) of group 1 cases completely recovered and were discharged from hospital, meanwhile 102 (9%) developed pulmonary and/or systemic complications. Mortality was as high as 18% in this group. On the contrary, group 2 cases showed a significantly higher recovery rate (87%) and lower complications, and mortality rate (3% and10%) respectively (*P* < 0.05; see Figure 1).

## Discussion

Community-acquired pneumonia (CAP) is one of the most common and serious infections in children, with an incidence of 34 to 40 cases per 1,000 in industrialized countries. In the developing world, CAP is even more common and more severe and is the largest killer of children [12]. Understanding the multiple social factors that affect the severity of CAP among Egyptian children is fundamental to planning and implementing effective health promotion actions in the country.

In our study, the median age of CAP patients was 5.4 years old which was higher than that found in previous studies on etiology of CAP in hospitalized children from Finland (median age 3.8 years), the USA (median age 33 months), and Norway (median age 23 months) [13-15].

Our data showed that a poor immunization record, a low birth weight, and an artificial feeding practice were more common among group 1 cases, with a statistically significant difference between the studied groups (all *P < 0.01*). These results are concordant with a recent Indian study which reported that younger age, a low birth weight, lack of breastfeeding, and a lower socioeconomic status (SES) are all associated with childhood pneumonia [16].

Poor children, especially in developing countries, are not only more exposed to environmental hazards but are also less likely to be covered by preventative interventions such as a vaccination policy. In our study, lack of breastfeeding was identified as being an important determinant associated with CAP among group 1 cases (*P < 0.01*). Previous studies reported that a lack of breastfeeding increases the risk of lower respiratory tract illnesses and development of severe pneumonia by 1.5 to 2.6 times [17]. In addition to passive protection, breast milk seems to positively affect the child’s systemic immune system via multiple mechanisms including immunomodulatory, maturational, anti-inflammatory, and antimicrobial actions [18].

As expected, we found that 304 (27%) of group 1 cases were malnourished compared to only 17 (5%) of the group 2 subjects (*P < 0.01*). Previous studies revealed that a lower SES and being malnourished increases the risk of pneumonia episodes [19,20].

In the present study, except for septicemia was not observed in group 2 cases, both findings from the history/background check (cough, difficulty breathing, chest pain) and findings from the physical examination (tachypnea, focal rales, focal wheezing, triage temperature, and oxygen saturation%) did not show significantly different results among the studied groups (all *P > 0.05*).

Our study showed that a young maternal age, a low maternal education level, parents’ smoking habits, rural residency, a low family income, and unavailability of adequate medical care were all significantly associated with the risk of severe CAP among the studied cases (all *P < 0.01*). Meanwhile, the parents’ occupation and housing conditions were not (*P > 0.05*).

Children with teenage mothers were more likely to have severe CAP compared to those born to older women (*P < 0.01*), a finding that is consistent with those reported by other authors [6]. This may be associated with older mothers having a better capacity for, and more experience with, childcare. Therefore, postponing childbirth and marriage until the age of 20 or later is important to improve child survival.

Parental smoking did have a definable impact on our figures and it remains a significant independent predictive risk factor for severe CAP among the studied groups (*P < 0.05*). Previous studies have shown that children whose parents smoke have a higher risk of contracting severe pneumonia and being hospitalized [21,22]. However, no studies have established a direct relationship between parents smoking and a higher occurrence of CAP-related complications [22].

Rural or urban residence did influence the severity of the disease in our study (*P < 0.01*). A likely reason is that in the East Nile Delta of Egypt—the locality of our study—the cities and villages are vastly different in terms of lifestyle, access to adequate medical care, sociocultural backgrounds, and literacy.

Surprisingly, we did not observe any significant relationship between housing conditions and severity of CAP among the studied subjects (*P > 0.05*), which was contrary to our expectations and to previous findings of Grant et al., who reported that lower quality living environments increase the risk of CAP and that improving housing quality may reduce the burden of CAP in New Zealand [23]. However, reporting in our study may have been unreliable, and a study to cover an extended population is recommended to better assess housing quality in the Nile Delta of Egypt, both in rural and urban areas.

Our multivariate logistic model indicated that a low maternal education level, unavailability of adequate medical care, a low family income, and parents’ smoking habits were significant independent predictive risk factors for severe CAP among the studied children (all *P < 0.05*).

Maternal education has emerged as an important determinant for the severity of CAP among the studied subjects (OR: 3.8; 95% CI: 2.12 –6.70; *P* = .0001). This is in agreement with a study conducted by Tiewsoh et al., who reported that prolonged hospital stay and need for mechanical ventilation were more common among CAP cases who had less-educated mothers [22]. A possible explanation may be that educated mothers identify illness much earlier in their children and avail early treatment. However, there are few reports linking maternal education level with the severity and outcome of childhood pneumonia.

In the present study, both unavailability of adequate medical care and a lower family income predicted a greater likelihood of having severe CAP (OR: 3.1; 95% CI: 1.99 –4.88; *P* = .0001 and OR: 2.2; 95% CI: 0.99 –4.78; *P* = .047, respectively). Chen et al. reported that a lower family income in the child**’**s early life predicted higher odds for having activity limitations and conditions that require physician treatment [5]. These observations could be due to the differences in the type of medical care provided for poor families who usually have limited access to adequate preventative and curative interventions. However, understanding an individual**’**s lifetime history of family income may be more important than understanding the current income conditions in terms of predicting children’s health [5].

Of note, CAP outcomes were significantly different between SES levels. Our data confirmed that higher complication and mortality rates existed among low SES (group 1) compared with high SES (group 2) (*P* < 0.05). These results are concordant with an earlier study conducted by Wood et al. who found an increased relative risk (RR 2.3 95% CI: 1.4–4.0) for lower social class quintiles and pneumonia mortality [24]. Similar studies conducted in Bangladesh suggested that beyond inter-country inequities, further critical inequities are present within countries, where children from the poorest families, those living in rural areas, and those whose mothers are less educated are more likely to die from severe pneumonia [25,26].

This dataset gives a unique insight into the burden that the SES of the patient and his/her family can have on CAP and on hospital admissions due to CAP in the East Nile Delta of Egypt. Since these social risk factors are potentially preventable, health policies targeted at reducing their prevalence could decrease the burden of childhood CAP. Nonetheless, extended survey studies that include additional measures of SES would allow us to test the generalizability of our findings more comprehensively.

## Conclusion

Public health measures against these sociodemographic risk factors should be taken and identified as priorities in order to help reduce the global burden of deaths from severe CAP among Egyptian children.

## Competing interests

The authors declare that they have no conflicts of interests.

## Authors’ contributions

SFA designed the study and the tools, collected the data, performed the statistical analysis, wrote the discussion, and submitted the manuscript. LS and SS reviewed the results and the discussion. WE and ME participated in the design of the study and helped draft the manuscript*.* SMA conceived the study, and coordinated the data collection and the analysis. All authors read and approved the final manuscript

## Supplementary Material

Additional file 1 Multilingual abstracts in the six official working languages of the United Nations. Click here for file
