# Supplementary material for: Impact of the socioeconomic status on the severity and outcome of community-acquired pneumonia among Egyptian children: a cohort study
Source: Infect Dis Poverty. 2014 Apr 24;3:14. doi: 10.1186/2049-9957-3-14 (PMC4022265; doi:10.1186/2049-9957-3-14)
Supplement: Additional file 1 — Multilingual abstracts in the six official working languages of the United Nations. [file 2049-9957-3-14-S1.pdf]

Translation of the abstract into the six official working languages of the United Nations

تأثير الوضع الاجتماعي والاقتصادي على شدة ونتائج الالتهاب الرئوي المكتسب من المجتمع بين الأطفال المصريين :دراسة استباقية

سهام عزب، ليلى محمد شريف، صفاء صالح، وفاء السعيد، منى الشافعي، سناء عبد السلام

الملخص

خلفية: يعد الالتهاب الرئوي المكتسب من المجتمع أحد أهم خمسة أسباب تتسبب في وفاة الأطفال في البلدان النامية، وهو ما يمثل ما يقرب من ثلاثة ملايين حالة وفاة سنويا. وتحديد عوامل الخطر القابلة للتعديل للالتهاب الرئوي المكتسب من المجتمع قد يساعد على الحد من عبء هذا المرض.

.

الهدف: قامت الدراسة ببحث تأثير الوضع الاجتماعي والاقتصادي على شدة ونتائج الالتهاب الرئوي المكتسب من المجتمع بين الأطفال المصريين.

الأساليب: تمت الدراسة من خلال عمل دراسة استباقية طولية شملت 1470 طفلا مصابين بالالتهاب الرئوي المكتسب من المجتمع، تتراوح أعمارهم بين سنتين و15 سنة (متوسط العمر 5.4 سنوات). واستند تشخيص الالتهاب الرئوي المكتسب من المجتمع على النتائج السريرية والإشعاعية. واستخدم استبيان منظم والسجلات الطبية للمرضى لجمع البيانات. وتم تقسيم الحالات موضوع الدراسة إلى مجموعتين تبعا لشدة الإصابة: خفيفة الإصابة وشديدة الإصابة. وتمت مقارنة المتغيرات الاجتماعية والديموغرافية، وعمل تحليل الانحدار اللوجستي متعدد المتغيرات.

وأظهر التحليل متعدد المتغيرات أن انخفاض مستوى تعليم الأمهات (OR:3.8; 95% CI:2.12–6.70; *P* = .0001)، وعدم توافر الرعاية الطبية الكافية (OR:3.1; 95% CI:1.99–4.88; *P* = .0001)، وانخفاض دخل الأسرة (OR:2.2; 95% CI:0.99 –4.78; *P* = .047)، وكون الآباء من المدخنين (OR:2.0; 95% CI:1.15–3.55; *P* = .014) عوامل خطر مستقلة تنبؤية هامة للالتهاب الرئوي المكتسب من المجتمع الشديد بين الأطفال المصريين.

الاستنتاج :ينبغي اتخاذ تدابير الصحة العامة ضد عوامل الخطر الاجتماعية والديموغرافية هذه باعتبارها أولويات من أجل المساعدة في تقليل العبء العالمي للوفيات الناجمة عن الالتهاب الرئوي المكتسب من المجتمع الشديد بين الأطفال المصريين.

Translated from English version into Arabic by Mahmoud Sami, through

TRANSLATORS
WITHOUT BORDERS

社会经济状况对埃及儿童社区获得性肺炎的严重性和结局的影响：队列研究

Seham F. A. Azab, Laila M. Sherief , Safaa H. Saleh, Wafaa F. Elsaeed, Mona A. Elshafie, Sanaa M. Abdelsalam

摘要：

引言：社区获得性肺炎是发展中国家儿童五大致死原因之一，每年导致大约300万死亡病例。确认那些可改变的危险因素将有助于降低该病的疾病负担。

目的：本研究探讨了社会经济状况对埃及儿童社区获得性肺炎的严重性和结局的影响。

方法：本前瞻性队列研究纳入了 1 470 名患有社区获得性肺炎的儿童，年龄在 2～15 岁之间（平均 5.4 岁）。社区获得性肺炎的诊断依据临床和影像学表现。通过结构化问卷和患者病历收集相关数据。将研究对象分为 2 组，即轻度组和重度组。采用多因素 logistic 回归分析相关社会和人口学因素的作用。

结果：母亲低教育水平、无法获得足够的医疗资源、低家庭经济收入、父母吸烟史是埃及儿童重度社区获得性肺炎的独立危险因素，其比值比（OR）分别为 3.8[95% 可信区间（CI）：2.12–6.70）]、3.1（95% CI：1.99–4.88）、2.2（95% CI：0.99–4.78）和 2.0（95% CI：1.15–3.55。

结论：应该将有助于改变以上社会人口学危险因素的公共卫生措施作为重点以降低埃及儿童重度社区获得性肺炎的疾病负担。

Translated from English version into Chinese by Qian Men-bao, through

CHINESE CENTER FOR DISEASE CONTROL AND PREVENTION
NATIONAL INSTITUTE FOR PARASITIC DISEASES

Impact du niveau socioéconomique sur la sévérité et les conséquences de la pneumonie communautaire chez l'enfant en Egypte : Etude de cohorte

Seham F. A. Azab, Laila M. Sherief, Safaa H. Saleh, Wafaa F. Elsaeed, Mona A. Elshafie, Sanaa M. Abdelsalam

RESUME

Contexte : La pneumonie communautaire compte parmi les cinq principales causes de mortalité chez l'enfant dans les pays en développement, responsable d'environ trois millions de décès chaque année. L'identification des facteurs de risques évitables de la pneumonie communautaire pourrait aider à réduire la portée de la maladie.

Objectif : Dans cette étude, nous avons mis en avant l'impact du niveau socioéconomique sur la sévérité et les conséquences de la pneumonie communautaire chez les enfants en Egypte.

Méthodes : Nous avons réalisé une étude prospective et longitudinale de cohorte incluant 1 470 enfants atteints de pneumonie communautaire âgés entre deux et 15 ans (âge médian : 5,4 ans). Le diagnostic de pneumonie communautaire a été posé suite à des examens cliniques et radiologiques. Un questionnaire structuré et le dossier médical des patients ont permis de récolter les données de l'étude. Les sujets ont été répartis en deux groupes : pneumonie communautaire modérée et pneumonie communautaire aigüe. Nous avons comparé les facteurs socio-démographiques et nous avons réalisé une analyse de régression logistique multivariée.

Résultats : L'analyse multivariée a démontré que le faible niveau de scolarité de la mère, (OR : 3,8 ; 95% , Ic ; 2.12–6.70; *P* = .0001), l'absence de soins médicaux appropriés (OR: 3,1 ; 95% , Ic ; 1.99–4.88; *P* = .0001), les faibles revenus des familles (OR: 2,2 ; 95% , Ic ; 0.99 –4.78; *P* = .047) et le tabagisme des parents (OR: 2,0 ; 95% , Ic ; 1.15–3.55; *P* = .014) sont d'importants facteurs de risque indépendants qui favorisent la pneumonie communautaire aigüe chez l'enfant en Egypte.

Conclusion : Les mesures de santé publique pour lutter contre ces facteurs de risque socio-démographiques doivent être identifiées en priorité afin d'aider à réduire le nombre de décès causés par la pneumonie communautaire aigüe chez l'enfant en Egypte.

Translated from English version into French by Fanny, through

TRANSLATORS
WITHOUT BORDERS

Влияние социально-экономического статуса на степень тяжести и исход внебольничной пневмонии среди египетских детей. Когортное исследование.

Сехам Ф. А. Азаб, Лэйла М. Шериф, Сафаа Х. Салех, Уафаа Ф. Эльсаид, Мона А. Эльшафи, Санаа М. Абдельсалам

РЕЗЮМЕ

История вопроса: Внебольничная пневмония (ВБП) является одной из пяти основных причин смерти среди детей в развивающихся странах и служит причиной около трех миллионов смертей в год. Распознание поддающихся изменению факторов риска может помочь сократить угрозу этой болезни.

Цель: В данном исследовании рассматривается влияние социально-экономического статуса (СЭС) на тяжесть и исход ВБП среди египетских детей.

Методы: Было проведено проспективное лонгитюдное когортное исследование, охватившее 1 470 детей с диагнозом ВБП в возрасте от двух до 15 лет (средний возраст – 5,4 года). Диагноз ВБП был основан на клинических и рентгенологических данных. Для сбора данных использовались специально разработанные вопросники и медицинские карты пациентов. Пациенты были разделены на две группы: пациенты с легкой и тяжелой формой ВБП. Было произведено сравнение социальных и демографических переменных, и проведен анализ путем множественной логистической регрессии.

Результаты: Множественный анализ показал, что низкий уровень образования матери (OR: 3,8; 95% CI: 2,12–6,70; *P* = 0,0001), недоступность адекватного медицинского обслуживания (OR: 3,1; 95% CI: 1,99–4,88; *P* = 0,0001), низкий доход семьи (OR: 2,2; 95% CI: 0,99 –4,78; *P* = 0,047) и родительские привычки к курению (OR: 2,0; 95% CI: 1,15–3,55; *P* = 0,014) являются значительными независимыми предсказующими факторами риска тяжелой формы ВБП среди египетских детей.

Заключение: Меры здравоохранения, направленные против этих социально-демографических факторов риска, необходимо сделать приоритетными в целях снижения глобальной угрозы смертей от тяжелой формы ВБП среди египетских детей.

Translated from English version into Russian by Elena McDonnell, through

TRANSLATORS
WITHOUT BORDERS

Impacto del estatus socioeconómico sobre la gravedad y el desenlace de la neumonía adquirida en comunidad entre los niños egipcios: Un estudio de cohorte

Seham F. A. Azab, Laila M. Sherief, Safaa H. Saleh, Wafaa F. Elsaeed, Mona A. Elshafie, Sanaa M. Abdelsalam

RESUMEN

Antecedentes: La neumonía adquirida en comunidad (CAP por su sigla en inglés) es una de las cinco principales causas de mortalidad entre los niños en países en desarrollo, lo cual explica las casi tres millones de muertes por año. La identificación de los factores de riesgo modificables de la CAP pueden ayudar a reducir el impacto de esta enfermedad.

Objetivos: En este estudio, se investigó el impacto del estatus socioeconómico (SES) sobre la gravedad y el desenlace de la CAP entre los niños egipcios.

Métodos: Este fue un estudio de cohorte longitudinal prospectivo que incluyó 1.470 niños diagnosticados con CAP, con edades entre 2 y 15 años (edad media 5,4 años). El diagnóstico de la CAP se basó en los hallazgos clínicos y radiológicos. Se usó un cuestionario estructurado y los registros médicos de los pacientes para la recolección de información. Los pacientes fueron divididos en dos grupos: CAP leve y severa. Se compararon las variables sociales y demográficas y se realizó un análisis de regresión logística multifactorial.

Resultados: El análisis multifactorial demostró que un bajo nivel de educación materna (OR [Índice de probabilidad]: 3,8; 95% CI [Intervalo de confianza]: 2,12–6,70; *P* = ,0001), falta de disponibilidad de cuidados médicos adecuados (OR: 3,1; 95% CI: 1,99–4,88; *P* = ,0001), bajo ingreso familiar (OR: 2,2; 95% CI: 0,99 –4,78; *P* = ,047) y tabaquismo de los progenitores (OR: 2,0; 95% CI: 1,15–3,55; *P* = ,014) fueron factores de riesgos predictivos independientes para la CAP severa entre los niños egipcios.

Conclusión: Se deberían identificar como prioritarias las medidas de salud pública a tomar contra estos factores de riesgo sociodemográfico para poder ayudar a reducir el impacto global de las muertes debidas a la CAP severa entre los niños egipcios.

Translated from English version into Spanish by Adriana Acevedo, through

TRANSLATORS
WITHOUT BORDERS
